# Revisiting the relationship between cardiorespiratory fitness and biological aging: insights from DunedinPACE analysis

**DOI:** 10.1007/s11357-025-01930-0

**Published:** 2025-10-30

**Authors:** Takuji Kawamura, Nobuhiro Nakamura, Hiroki Tabata, Ryoko Kawakami, Chiyoko Usui, Tomoko Ito, Keishi Soga, Yasuyuki Taki, Zsolt Radak, Suguru Torii, Katsuhiko Suzuki, Kaori Ishii, Shizuo Sakamoto, Motohiko Miyachi, Koichiro Oka, Mitsuru Higuchi, Isao Muraoka, Kumpei Tanisawa

**Affiliations:** 1 Smart Aging Research Center, Tohoku University, 4-1 Seiryo-Machi, Aoba-Ku, Sendai, Miyagi 980-8575 Japan; 2 Faculty of Sport Sciences, Waseda University, Saitama, 359-1192 Japan; 3 Waseda Institute for Sport Sciences, Waseda University, Saitama, 359-1192 Japan; 4 Sportology Center, Juntendo University Graduate School of Medicine, Tokyo, 113-0034 Japan; 5 Physical Fitness Research Institute, Meiji Yasuda Life Foundation of Health and Welfare, Tokyo, 192-0001 Japan; 6 Center for Liberal Education and Learning, Sophia University, Tokyo, 102-8544 Japan; 7 Research Center for Molecular Exercise Science, Hungarian University of Sports Science, Budapest, 1123 Hungary

**Keywords:** Epigenetic aging, DunedinPACE, Cardiorespiratory fitness, Biological aging, Epigenetic clock, Receiver operating characteristic curve analysis

## Abstract

DunedinPACE quantifies the pace of biological aging. No study has examined its association with cardiorespiratory fitness (CRF). Additionally, the physiologically relevant CRF thresholds associated with slow aging remain unclear. The purpose of this study was to investigate the association between CRF and the pace of epigenetic aging, as measured by DunedinPACE, and to identify a CRF threshold indicative of slower biological aging. Here, we analyzed data of 144 older men (aged 65–72 years) enrolled in the WASEDA’S Health Study to examine the association of CRF (VO₂/kg at peak and ventilatory threshold (VT)), physical fitness, anthropometric parameters, and nutritional intake with epigenetic aging. Epigenetic aging was assessed using DunedinPACE derived from blood DNA methylation profiles. We performed Pearson’s and partial correlation analyses adjusted for age, smoking status, and drinking status, followed by receiver operating characteristic (ROC) curve analyses with bootstrapped optimal cutoff determination. In the unadjusted analyses, VO₂/kg at peak (*r* =  − 0.17, *p* = 0.041) and VT (*r* =  − 0.17, *p* = 0.046) were inversely associated with DunedinPACE, and VO₂/kg at peak showed a significant association even in the adjusted models (*r* =  − 0.16, *p* = 0.046). The ROC curve analysis revealed a potential threshold of VO₂/kg at peak (26.2 mL/kg/min) for differentiating individuals with slower biological aging, supported by bootstrap distributions of optimal cutoff points and Youden’s index. This study suggests that higher CRF is associated with a slower pace of epigenetic aging, as measured using DunedinPACE. The identified VO₂peak threshold may provide a biomarker-based fitness target to support healthy aging in older adults.

**Supplementary Information:**

The online version contains supplementary material available at https://doi.org/10.1007/s11357-025-01930-0.

## Introduction

The increase in medical and healthcare costs and the decline in the quality of life associated with population aging are major global issues, and strategies to extend healthy life expectancy are required. One promising approach is to develop biomarkers that estimate an individual’s biological age rather than the chronological age, as such measures can detect accelerated aging earlier and allow for targeted lifestyle or clinical interventions aimed at preserving function and potentially extending healthy life expectancy [1]. Although the traditional measures include telomere length and various blood biomarkers, DNA methylation-based epigenetic clocks have recently gained considerable attention. These clocks calculate an individual’s biological age by summing the methylation levels at specific CpG sites in the genome. The difference between biological age estimated by these clocks and chronological age is often referred to as epigenetic age acceleration. Notably, accelerated epigenetic age has been associated with increased risk of all-cause mortality, as well as higher incidence of age-related diseases including cardiovascular disease, cancer, and cognitive decline [2, 3].

Dunedin Pace of Aging Calculated from the Epigenome (DunedinPACE), a new indicator derived from methylation levels, shifts the focus from estimating biological age in a cross-sectional manner to measuring the rate at which aging progresses [4]. Individuals with a faster pace of aging exhibit higher DunedinPACE values, whereas those with a slower pace of aging exhibit lower values [4]. Given its ability to capture longitudinal changes in biological aging, DunedinPACE has emerged as a powerful tool in epidemiological and interventional contexts for examining how lifestyle factors influence aging rates. Indeed, accumulating evidence indicates that DunedinPACE is a sensitive biomarker of biological aging that responds to a range of modifiable lifestyle and environmental factors, including socioeconomic disadvantages, smoking habit, obesity, diet quality, and physical activity [4–8]. Furthermore, recent interventional trials have suggested that caloric restriction and omega-3 supplementation may reduce DunedinPACE value, indicating its potential responsiveness to health-promoting behavioral changes [9, 10]. These findings suggest that DunedinPACE serves as a dynamic biomarker that reflects biological damage and resilience throughout life.

Cardiorespiratory fitness (CRF), traditionally quantified through direct measurement of maximal oxygen uptake (VO₂max), has been established to play a crucial role in reducing mortality and cardiometabolic risk [11, 12]. However, only a limited number of studies have investigated the relationship between CRF and epigenetic aging. In this context, we previously investigated the relationship between direct gas exchange measurements of CRF and multiple epigenetic clocks in older men, revealing a negative association between CRF and epigenetic age acceleration (EAA). The finding suggests that individuals with higher CRF may experience slower epigenetic aging [13]. Specifically, in cross-sectional data, peak oxygen uptake (VO₂peak) and oxygen uptake at the ventilatory threshold (VT) showed a negative correlation with GrimAgeAccel [13]. In addition to CRF, we found that epigenetic clocks may reflect nutritional and metabolic status. For instance, micronutrients such as copper, iron, and β-carotene, as well as high-density lipoprotein cholesterol (HDL-C) and visceral fat area, have been shown to correlate with phenoAgeAccel, GrimAgeAccel, and FitAgeAccel [13]. These results imply that maintaining and improving physical fitness through regular exercise, as well as optimizing nutritional and metabolic profiles, could serve as strategic means to delay epigenetic aging.

Despite these implications, the specific association between CRF and epigenetic measures of the pace of aging, such as DunedinPACE, has not yet been investigated, and how exercise may regulate the rate of aging remains elucidated. Therefore, building on our previous work [13], in this study, we aimed to further explore whether CRF is associated with epigenetically assessed pace of aging, as measured using DunedinPACE, within the framework of the WASEDA’S Health Study. To further refine the evaluation of CRF thresholds, we employed bootstrap-based receiver operating characteristic (ROC) curve analysis, which provides robust estimates of optimal cutoff points and their stability across resampled datasets [14]. Thus, we provide insights for establishing exercise strategies to modulate the pace of aging and promote healthy longevity.

## Materials and methods

### Participants

This study was conducted using a previously reported method, targeting participants of the Waseda Alumni’s Sports, Exercise, Daily Activity, Sedentariness and Health Study (WASEDA’s Health Study) as described previously [13, 15–22]. This study involved 144 older men selected from Cohort D, for whom valid DNA methylation and CRF data were available. Details of Cohorts A–D in the WASEDA’S Health Study can be found elsewhere [18]. In this study, we focused on phenotypic variables previously reported to be associated with epigenetic clocks [13]. All participants provided written informed consent before the measurements and surveys. This study was approved by the Research Ethics Committee of Waseda University (approval numbers: 2014-G002 and 2018-G001) and conducted in accordance with the tenets of the Declaration of Helsinki.

### Anthropometry, nutrition, physical fitness, and lifestyle assessments

Anthropometric and body composition parameters, including height, weight, body mass index (BMI), body fat percentage, fat-free mass, visceral and subcutaneous fat areas, and abdominal and calf circumference, were assessed using previously described methods (13). Briefly, body composition was evaluated using bioelectrical impedance analysis, and fat distribution was quantified using magnetic resonance imaging (MRI). CRF was measured using a graded exercise test on a cycle ergometer with direct breath-by-breath gas analysis, calculating VO₂ at the VT and VO₂peak (absolute and relative to body weight). Muscle function was assessed by measuring handgrip strength and leg extension power. Nutritional intake was estimated using a validated brief-type self-administered diet history questionnaire (BDHQ), with nutrient intake values calculated per 1000 kcal. Lifestyle information, including smoking status (never, former, and current), drinking status (less than once a week, 2–4 times a week, and more than 5 times a week), and chronotype (based on the Morningness–Eveningness Questionnaire [MEQ]), was collected via structured questionnaires.

### Blood collection and biochemical analyses

Blood samples were collected and processed using a previously described protocol [13, 18, 21]. Briefly, the participants fasted overnight (≥ 12 h) before blood collection. Venous blood was drawn into tubes with or without an anticoagulant (EDTA-2Na with sodium fluoride, or heparin sodium) and centrifuged at 4 °C for 10 min at 3000 rpm. The resulting serum were aliquoted and stored at − 80 °C until analysis. Serum triglyceride, total cholesterol, HDL-C, and low-density lipoprotein cholesterol (LDL-C) were also quantified. All biochemical analyses were performed by BML, Inc. (Tokyo, Japan).

### DNA methylation profiling and epigenetic age estimation

Genomic DNA was extracted from the whole blood collected in EDTA-2 K tubes using a QIAamp DNA Midi Kit (Qiagen, Hilden, Germany), as previously described [13, 21]. Briefly, DNA samples were dissolved in Buffer AE (≥ 50 ng/μL), with A260/280 ratios between 1.7 and 2.1. Genome-wide DNA methylation was assessed using an Infinium MethylationEPIC BeadChip Kit (Illumina Inc.), followed by bisulfite conversion using an EZ DNA Methylation Kit (Zymo Research). Quality control was performed using R (version 4.0.5) with the minfi, Meffil, and ewastools packages. The samples of all 144 participants met the quality control criteria. Based on the methylation data, five epigenetic clocks, Horvath [23], Hannum [24], PhenoAge [25], GrimAge [26], and FitAge [27], were computed following established methods. For each clock, EAA (AgeAccel) was calculated as the difference between DNA methylation-based age (DNAmAge) and chronological age. DunedinPACE, a measure of the pace of aging, was also derived from methylation profiles as previously described [4].

### Statistical analysis

All statistical analyses were conducted using the R software (version 4.3.0; R Core Team, 2023). Pearson’s correlation coefficients were calculated for all epigenetic age indices, including DNAmAge (Horvath, Hannum, PhenoAge, GrimAge, and FitAge) and DunedinPACE, to assess the degree of shared variance and potential complementarity or divergence among the different clocks. We also examined the association between DunedinPACE and continuous variables, including anthropometric, nutritional, physical fitness, and biochemical measures. We performed Pearson’s correlation analysis, followed by partial correlation analyses adjusted for age, smoking status (never, former, or current), and drinking status to control for potential confounding factors. Given our objective to evaluate pure correlations rather than predictive relationships, a partial correlation analysis was employed to control confounding factors while preserving bidirectional relationships. These analyses aimed to explore the strength and direction of the associations between DunedinPACE and key physiological or lifestyle variables. In this study, slower biological aging was defined as DunedinPACE < 1.00. An ROC curve analysis with 2000 bootstrap replicates was performed to identify the optimal cutoff value of VO₂/kg at peak for predicting lower DunedinPACE values, indicative of slower biological aging. Performing 2000 bootstrap replicates is the standard practice for achieving stable and reliable confidence intervals, possibly leading to more defensible conclusions. The area under the curve (AUC) was calculated as a measure of predictive accuracy, and Youden’s index was used to determine the optimal threshold. To visualize and compare the phenotypic correlation patterns across epigenetic clocks, radar charts were constructed based on the absolute values of the partial correlation coefficients between each clock and 17 physiological and lifestyle-related variables. In addition, the enclosed area within each radar plot was calculated as an integrated indicator of the multidimensional association strength for each clock. Specifically, the area was computed by treating the radar chart as a regular polygon, with each axis representing the absolute partial correlation coefficient between a given epigenetic clock and one of the 17 phenotypic variables. The coordinates of each point on the radar were used to calculate the polygon’s area using the shoelace formula (also known as the surveyor’s formula), implemented in R. This area serves as a quantitative summary of how broadly and strongly each epigenetic clock is associated with a multidimensional phenotypic profile.

## Results

### Characteristics of the participants

The characteristics of the participants have been described previously [13]. Online Resource 1 presents the descriptive statistics for all participants (*n* = 144), who were Japanese men aged 65–72 years (mean age: 68.0 ± 1.9 years). The average VO₂/kg at peak was 26.5 ± 4.6 mL/kg/min and VO₂/kg at VT was 15.3 ± 3.4 mL/kg/min. The mean DunedinPACE value was 1.01 ± 0.09. Other variables, including anthropometric parameters, physical fitness, biochemical parameters, and nutritional intake, were consistent with normative data for healthy older adults. The categorical variables are summarized in Online Resource 2. In brief, 30.6% of the participants were non-smokers, 62.5% were past smokers, and 6.9% were current smokers. Regarding drinking status, 28.5% of the participants drank 0–1 time/week, 18.7% drank 2–4 times/week, and 52.8% drank 5–7 times/week. Regarding chronotype, 58.9% of the participants reported moderate and obvious morning type and 41.1% reported intermediate and moderate evening type, as previously reported [13].

### Correlations between epigenetic clocks

**Fig. 1:**
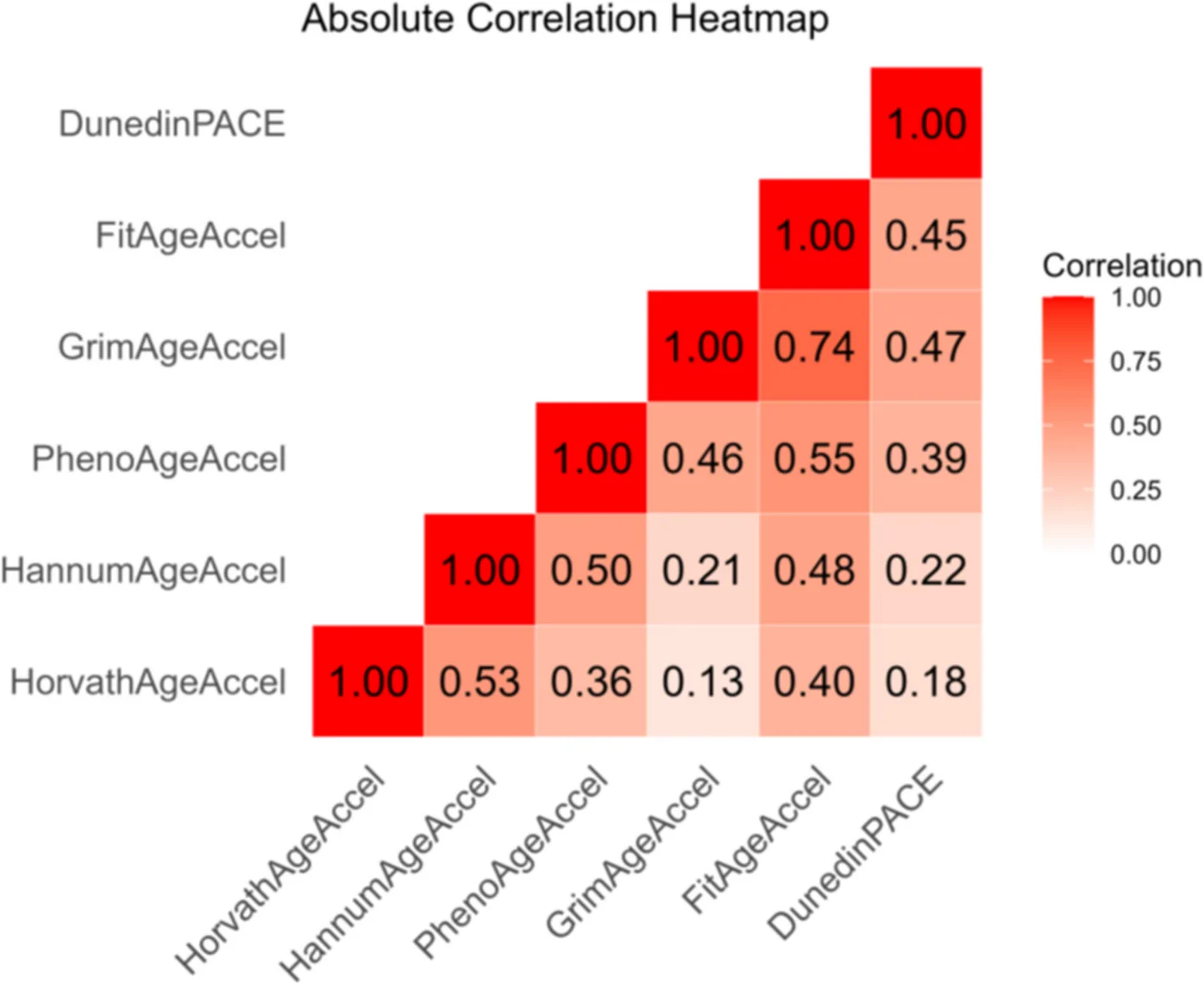
Peason’s correlations among epigenetic clocks. Pearson’s correlation coefficients were calculated for six epigenetic clocks: HorvathAgeAccel, HannumAgeAccel, PhenoAgeAccel, GrimAgeAccel, FitAgeAccel, and DunedinPACE. The color scale represents the strength of the correlations, with red indicating stronger positive relationships. Abbreviations: DNAmAge DNA methylation-based age, PACE Pace of Aging Calculated from the Epigenome

Figure 1 shows the correlations between epigenetic clocks. The Pearson’s correlation analysis revealed weak to strong correlations among the six DNA methylation-based age measures. DunedinPACE showed moderate correlations with the second-generation clocks PhenoAgeAccel (*r* = 0.39, *p* < 0.001), GrimAgeAccel (*r* = 0.47, *p* < 0.001), and FitAgeAccel (*r* = 0.45, *p* < 0.001), and weak correlations with the first-generation clocks HorvathAgeAccel (*r* = 0.18, *p* = 0.036) and HannumAgeAccel (*r* = 0.22, *p* = 0.009).

### Correlations between continuous variables and DunedinPACE

**Fig. 2:**
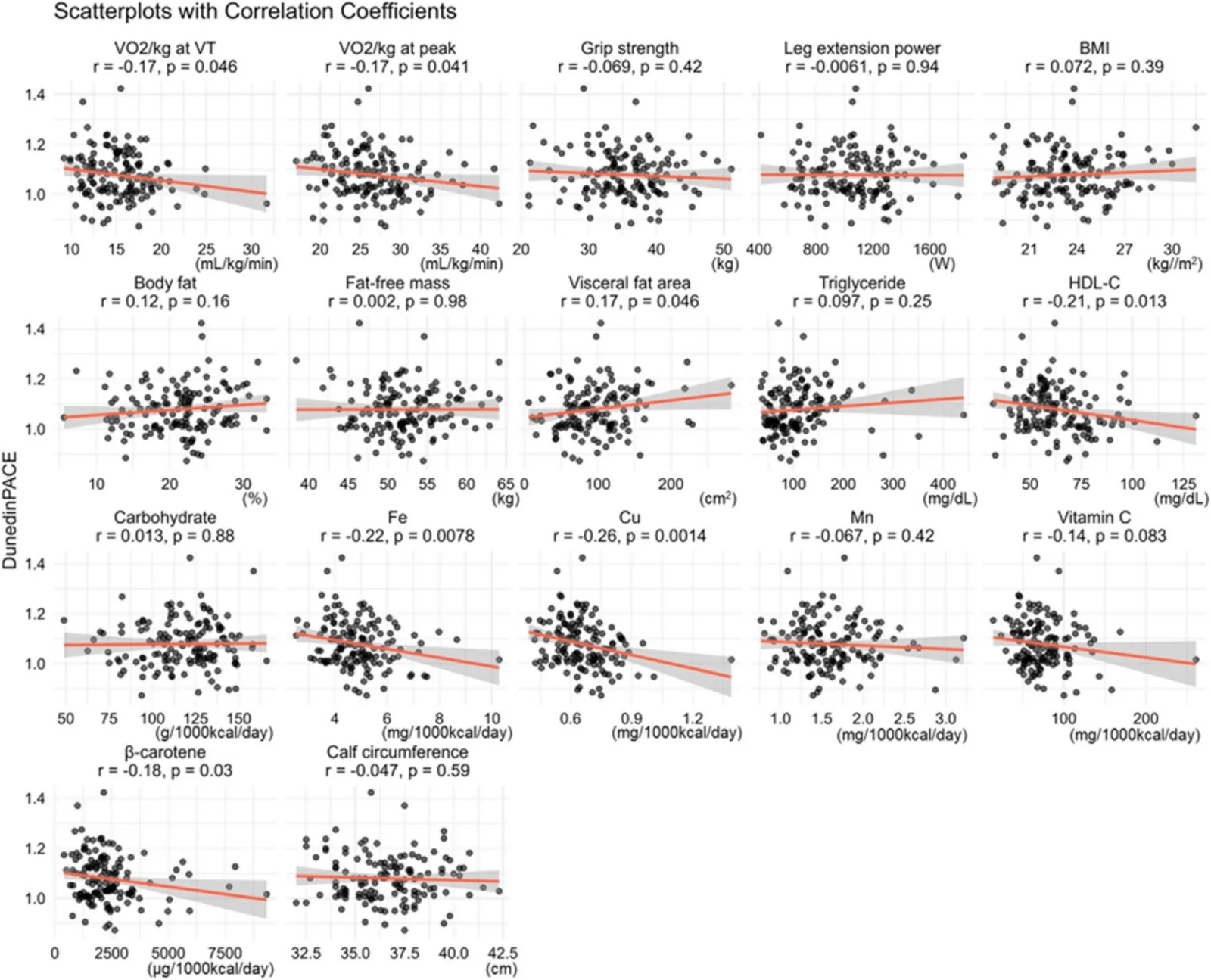
Pearson’s correlations between DunedinPACE and physiological, nutritional, and biochemical variables. Pearson’s correlation coefficients were calculated between DunedinPACE and a selected physiological, biochemical, or nutritional variable. Pearson’s correlation coefficients (*r*) and *p* values are shown in each subplot. The shaded gray region indicates the 95% confidence interval. DunedinPACE values reflect the pace of biological aging, where values > 1 indicate accelerated aging. Abbreviations: BMI body mass index, Cu copper, Fe iron, HDL-C high-density lipoprotein cholesterol, Mn manganese, PACE Pace of Aging Calculated from the Epigenome, VO₂/kg oxygen uptake per body mass

Figure 2 shows a scatter plot matrix between the continuous variables and DunedinPACE. The initial Pearson’s correlation analysis indicated that higher VO₂/kg at peak (*r* =  − 0.17, *p* = 0.041) and VO₂/kg at VT (*r* =  − 0.17, *p* = 0.046) were significantly associated with lower DunedinPACE values. Among anthropometric, nutritional, and biochemical parameters, DunedinPACE showed significant negative correlations with Cu (*r* =  − 0.26, *p* = 0.0014), Fe (*r* =  − 0.22, *p* = 0.0078), HDL-C (*r* =  − 0.21, *p* = 0.013), and β-carotene levels (*r* =  − 0.18, *p* = 0.03), and a positive correlation with visceral fat area (*r* = 0.17, *p* = 0.046). There was a significant trend toward a negative association between vitamin C level and DunedinPACE (*r* =  − 0.14, *p* = 0.083).

### Partial correlations between contentious variables and DunedinPACE

**Fig. 3:**
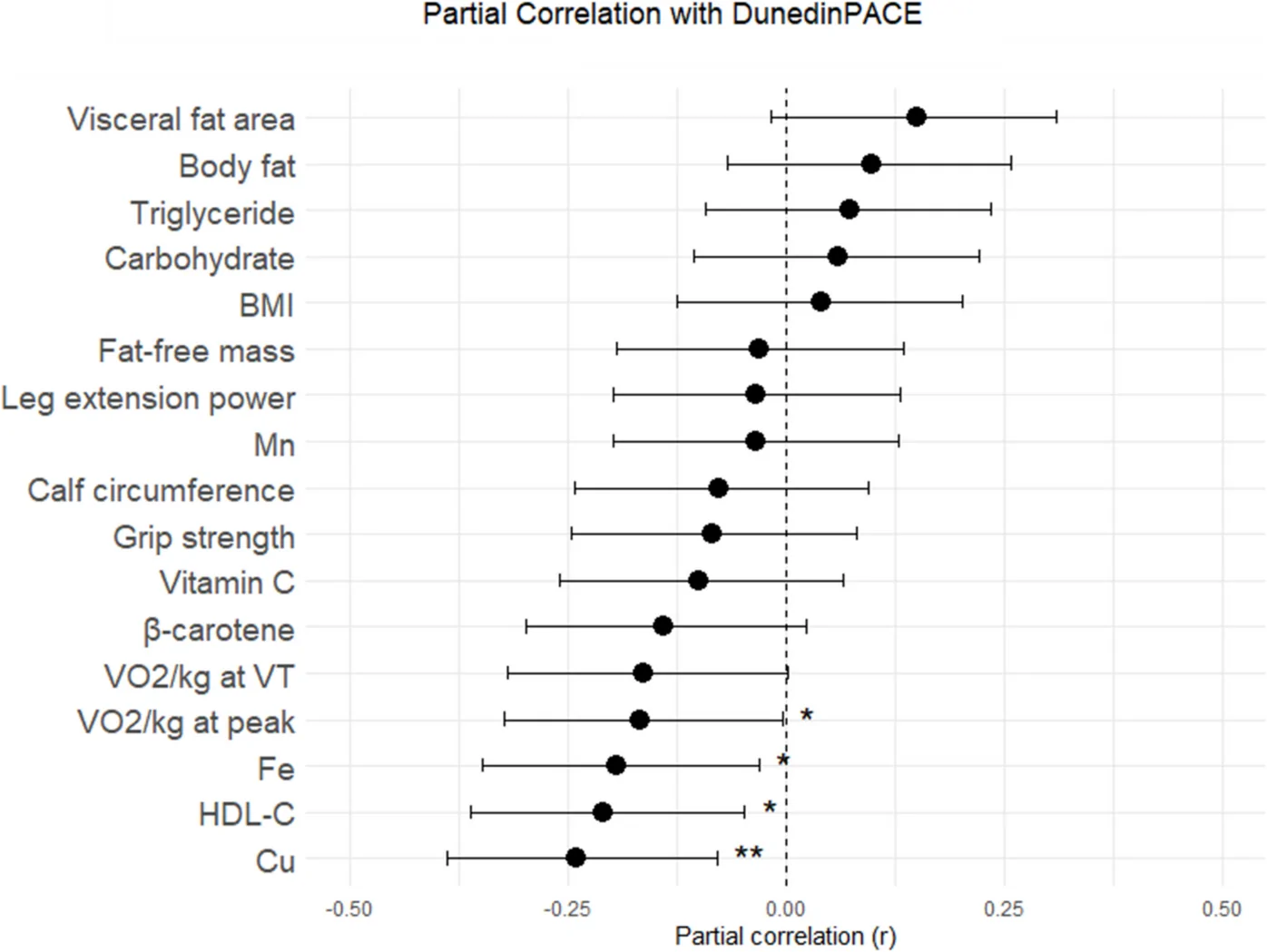
Partial correlations between DunedinPACE and selected variables. Partial correlation coefficients (adjusted for age, smoking status, and drinking status) were calculated between DunedinPACE and 17 variables related to physical fitness, body composition, nutrient intake, and biochemical parameters. The error bars represent 95% confidence intervals. Asterisks indicate significance levels (*p < 0.05; **p < 0.01). Abbreviations: BMI body mass index, Cu copper, Fe iron, HDL-C high-density lipoprotein cholesterol, Mn manganese, PACE Pace of Aging Calculated from the Epigenome, VO₂/kg oxygen uptake per body mass

Figure 3 shows the results of the partial correlation analyses. After adjusting for chronological age, smoking status, and drinking status, the partial correlation analysis revealed that VO₂/kg at peak remained significantly and inversely correlated with DunedinPACE (*r* =  − 0.17, *p* = 0.046). Serum Cu level showed the strongest negative association (*r* =  − 0.24, *p* = 0.004), followed by HDL-C (*r* =  − 0.21, *p* = 0.012) and Fe levels (*r* =  − 0.24, *p* = 0.004). Additionally, VO₂/kg at VT (*r* =  − 0.16, *p* = 0.054), β-carotene level (*r* =  − 0.14, *p* = 0.094), and visceral fat area (*r* = 0.15, *p* = 0.082) exhibited a significant trend of association with DunedinPACE.

### Bootstrapped optimal cutoff determination

**Fig. 4:**
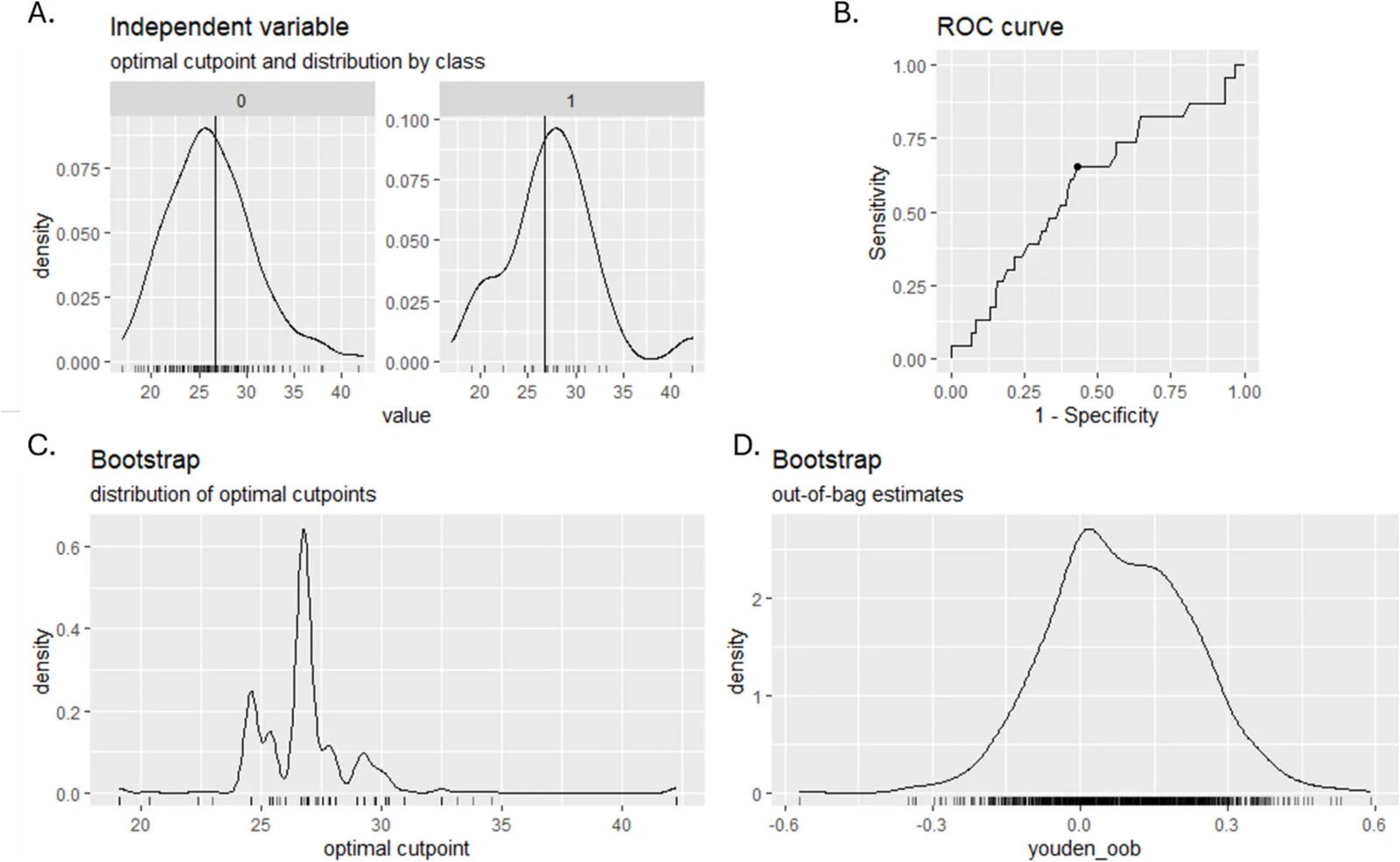
ROC analysis identifying the optimal cardiorespiratory fitness threshold for slower pace of biological aging. Receiver operating characteristic (ROC) curve analysis with 2000 bootstrap replicates was used to determine the optimal cutoff value of VO₂/kg at peak for classifying individuals with slower biological aging (DunedinPACE < 1.00). The area under the curve (AUC) and Youden’s index were used to evaluate classification performance. **a** The distribution of VO₂/kg at peak is shown separately for individuals with faster (class 0) and slower (class 1) biological aging, with the optimal cutoff point indicated with a vertical line in each panel. **b** The ROC curve depicts the trade-off between sensitivity and 1 − specificity, with the black point marking the optimal threshold that maximizes Youden’s index. **c** The distribution of optimal cutoff points across 2000 bootstrap replicates reflects the variability and robustness of the estimated threshold. **d** Distribution of out-of-bag estimates of Youden’s index demonstrates the cross-validated performance of the classifier

ROC curve analysis and bootstrapping (*n* = 2000 replicates) were conducted to explore the physiologically meaningful threshold of CRF for predicting slower aging. The optimal cutoff of VO₂/kg at peak for classifying lower DunedinPACE values was determined to be 26.2 mL/kg/min, based on Youden’s index, as shown in Fig. 4. The AUC was 0.69, with discriminative ability approaching the moderate range. This threshold was consistent across the bootstrap iterations, confirming its robustness.

### Phenotypic correlation profiles of epigenetic clocks

**Fig. 5:**
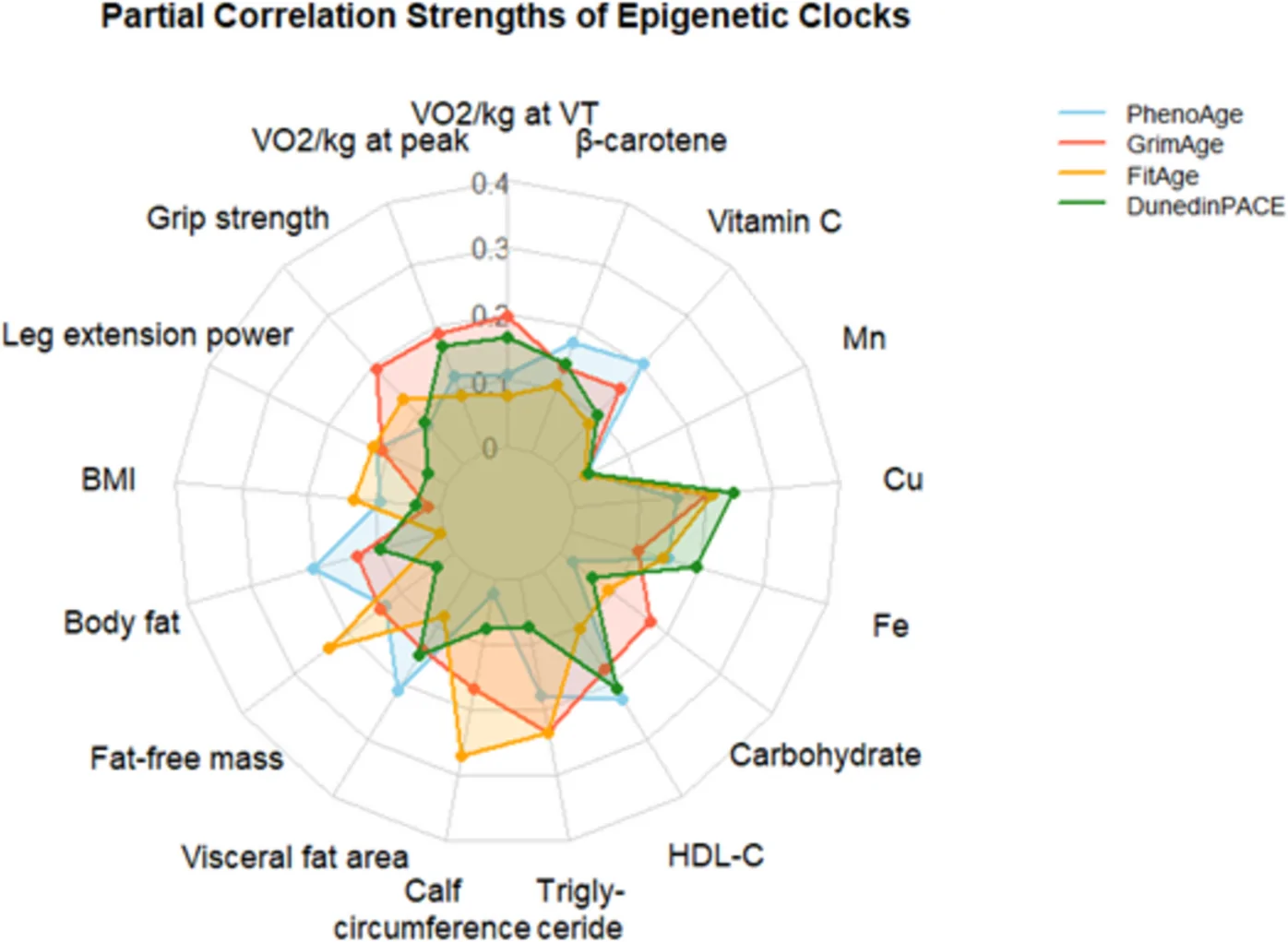
Radar chart of absolute partial correlation coefficients between epigenetic clocks and phenotypic variables. Each line represents the profile of absolute partial correlation coefficients (adjusted for age, smoking status, and drinking status) between one epigenetic clock and the 17 phenotypes. The shaded areas represent the integrated association strength of each clock. Abbreviations: BMI body mass index, Cu copper, Fe iron, HDL-C high-density lipoprotein cholesterol, Mn manganese, PACE Pace of Aging Calculated from the Epigenome, VO₂/kg oxygen uptake per body mass

To compare the multidimensional associations between epigenetic aging indices and physiological and lifestyle-related phenotypes, radar charts were generated using the absolute values of the partial correlation coefficients adjusted for age, smoking status, and drinking status in Fig. 5. These charts visually highlight the distinct phenotypic correlation profiles of the six DNA methylation-based aging measures: HorvathAgeAccel, HannumAgeAccel, PhenoAgeAccel, GrimAgeAccel, FitAgeAccel, and DunedinPACE. The enclosed area of each radar plot was calculated to quantify the overall strength of the association. The largest area was observed for GrimAgeAccel (0.0665), followed by PhenoAgeAccel (0.0474), FitAgeAccel (0.0474), HannumAgeAccel (0.0429), DunedinPACE (0.0380), and HorvathAgeAccel (0.0127).

## Discussion

This study suggests that higher CRF, as assessed using direct measurements of VO₂/kg at peak and VT, is associated with a slower pace of aging, as measured using DunedinPACE. To the best of our knowledge, this is the first study to propose a physiologically meaningful CRF threshold of 26.2 mL/kg/min for VO₂/kg at peak, which may aid in distinguishing older men with a slower rate of biological aging based on epigenetic profiling.

DunedinPACE has emerged as a second-generation epigenetic biomarker that captures the rate of aging. Our results showed that DunedinPACE correlates only weakly with the first-generation epigenetic clocks (namely, HorvathAgeAccel and HannumAgeAccel) and moderately with second-generation clocks (namely, PhenoAgeAccel, GrimAgeAccel, and FitAgeAccel). These results are reasonable, considering that first-generation clocks are advantageous for estimating chronological age, while second-generation clocks are advantageous for estimating biological age [1, 28].

In this study, the inverse correlation between CRF and DunedinPACE remained significant even after adjusting for age, smoking status, and drinking status. These results extend those of previous studies, including our study [13, 29], showing an association between high CRF and delayed epigenetic aging. In this study, we defined DunedinPACE < 1.00 as slow aging, since 1.00 represents the normative rate of aging (one biological year per chronological year), and values below this threshold indicate a slower pace of aging [4]. Interestingly, VO₂/kg at peak remained significantly associated with DunedinPACE after adjustment, whereas VO₂ at VT did not. This difference may be explained by the distinct physiological characteristics captured by these two parameters: VO₂peak reflects the maximal integrative capacity of the cardiovascular, pulmonary, and muscular systems [30], whereas VO₂ at VT represents a submaximal metabolic threshold reflecting the balance between aerobic and anaerobic energy supply [31]. Therefore, VO₂peak may serve as a robust measure of the integrative cardiorespiratory and muscular capacity that underpins physiological function in relation to epigenetic aging. In addition to CRF, several nutritional and biochemical factors inversely correlated with DunedinPACE in this study. Notably, there was a negative correlation with serum Cu and β-carotene levels, which are associated with oxidative stress pathways [32]. Fe and vitamin C levels also showed a significant trend towards negative correlations with DunedinPACE. These results are consistent with the hypothesis that the redox balance affects the aging process, suggesting that lifestyle-based interventions may be an effective strategy for slowing the pace of aging.

In this study, we employed a bootstrap-based ROC curve analysis to identify a CRF threshold that is effective in slowing the pace of aging. This method allows for a robust estimation of the optimal cutoff point and its stability across resampled datasets, thereby reducing the risk of overfitting and enhancing the generalizability of the findings [14]. Considering that 22.7 mL/kg/min represents the normative reference value for VO₂peak in men of similar age [33], the cutoff value determined in this study suggests that maintaining relatively high CRF is important for slowing the pace of aging. Our findings indicate that the incorporation of biological aging biomarkers into clinical or public health fitness assessments may contribute to the development of evidence-based guidelines for achieving healthy aging.

Although the observed correlation coefficients were relatively small (*r* =  − 0.17) and the discriminative ability of the ROC curve analysis was modest (AUC = 0.69), prior studies have demonstrated that even small differences in DunedinPACE are associated with meaningful differences in health outcomes, including morbidity and mortality risk at the population level [4, 34]. Therefore, while the clinical utility for individual prediction may be limited, these modest associations still provide valuable insights for understanding the potential role of CRF in slowing biological aging and for informing public health strategies.

To further explore the phenotypic correlations of epigenetic aging, we visualized the partial correlation profiles between the 6 clocks and 17 physiological and lifestyle-related variables using radar charts. The results revealed that GrimAgeAccel had the largest enclosed area, indicating the broadest phenotypic association, followed by PhenoAgeAccel and FitAgeAccel. In contrast, HorvathAgeAccel showed the narrowest profile. DunedinPACE exhibited moderate associations, particularly with fitness- and nutrition-related traits such as VO₂/kg at peak, serum copper level, and β-carotene level. These findings indicate the differential sensitivity of each clock to specific aging-related phenotypes and support the use of multidimensional metrics to interpret epigenetic biomarkers.

This study had certain limitations. We used a cross-sectional design in which both DNA methylation and CRF were measured only at a single time point; thus, we could not evaluate longitudinal trajectories, and causality could not be concluded. This implies that the potential for reverse causality cannot be excluded; that is, individuals with slower biological aging may have a greater capacity to achieve higher levels of CRF. In addition, the sample consisted of relatively healthy older Japanese men, limiting the generalizability to women, younger populations, or other ethnic groups. Furthermore, although DunedinPACE has demonstrated high predictive validity across multiple cohorts, its sensitivity to longitudinal changes in lifestyle and physical fitness needs to be confirmed in interventional studies. Despite these limitations, the findings of this study contribute to the growing body of evidence linking physical fitness and biological aging, and provide the first insights into specific CRF thresholds that slow the pace of epigenetic aging. Future studies should verify whether exercise interventions that improve CRF causally reduce DunedinPACE values or other epigenetic aging marker levels and whether these effects result in specific extensions of healthy life expectancy.

From a public health and clinical perspective, identifying physiologically meaningful CRF thresholds associated with slower biological aging has potential implications. Such thresholds may serve as practical targets for exercise prescriptions and fitness assessments in older adults. Although further validation in longitudinal and interventional studies is required, the incorporation of CRF thresholds into health promotion strategies could help translate epigenetic aging research into actionable approaches to support healthy longevity.

## Supplementary Information

Below is the link to the electronic supplementary material.

ESM 1 (CSV 17.1 KB)

ESM 2 (XLSX 9.39 KB)

## Data Availability

The datasets presented in this article are not readily available because the study is ongoing. The data used to support the results of this study are available from the corresponding author on reasonable request.
